# Bacterium-like particles as multi-epitope delivery platform for *Plasmodium berghei *circumsporozoite protein induce complete protection against malaria in mice

**DOI:** 10.1186/1475-2875-11-50

**Published:** 2012-02-20

**Authors:** Krystelle Nganou-Makamdop, Maarten L van Roosmalen, Sandrine AL Audouy, Geert-Jan van Gemert, Kees Leenhouts, Cornelus C Hermsen, Robert W Sauerwein

**Affiliations:** 1 Department of Medical Microbiology, Radboud University Nijmegen Medical Centre, P. O. Box 9101, 6500 HB Nijmegen, The Netherlands; 2 Mucosis BV, Groningen, The Netherlands; 3 BiOMaDe Technology Foundation, Groningen, The Netherlands

**Keywords:** BLP, CSP, Delivery platform, Immunization, Malaria, *Plasmodium berghei*

## Abstract

**Background:**

Virus-like particles have been regularly used as an antigen delivery system for a number of *Plasmodium *peptides or proteins. The present study reports the immunogenicity and protective efficacy of bacterium-like particles (BLPs) generated from *Lactococcus lactis *and loaded *with Plasmodium berghei *circumsporozoite protein (PbCSP) peptides.

**Methods:**

A panel of BLP-PbCSP formulations differing in composition and quantity of B-cell, CD4+ and CD8+ T-cell epitopes of PbCSP were tested in BALB/c mice.

**Results:**

BLP-PbCSP1 induced specific humoral responses but no IFN-γ ELISPOT response, protecting 30-40% of the immunized mice. BLP-PbCSP2, with reduced length of the non-immunogenic part of the T-cell-epitopes construct, increased induction of IFN-γ responses as well as protection up to 60-70%. Compared to controls, lower parasitaemia was observed in unprotected mice immunized with BLP-PbCSP1 or 2, suggestive for partial immunity. Finally, further increase of the number of B-cell epitopes and codon optimization (BLP-PbCSP4) induced the highest anti-CSP antibody levels and number of IFN-γ spots, resulting in sterile immunity in 100% of the immunized mice.

**Conclusion:**

Presentation of *Plasmodium*-derived antigens using BLPs as a delivery system induced complete protection in a murine malaria model. Eventually, BLPs have the potential to be used as a novel versatile delivery platform in malaria vaccine development.

## Background

By 2009, nearly a quarter of a billion people worldwide suffered from a malaria infection that resulted in approximately 800,000 deaths each year, mainly of children in sub-Saharan Africa [1]. Long-term solutions to stop deaths caused by malaria include the development of a prophylactic vaccine. Pre-erythrocytic stages of the parasite have been the principle target for vaccine development [2]. Effective delivery systems are required to optimize immune responses and protection by sub-unit based vaccines [3]. As such, virus-like particles (VLPs) have emerged as promising candidates able to induce cell-mediated immunity [4]. Due to its abundant presence on the sporozoite's surface [5], the circumsporozoite protein (CSP) has been the prime target for pre-erythrocytic protein-based malaria vaccine development [6-9]. In the *Plasmodium berghei *murine model, CSP immunizations with virally vectored delivery systems have been to shown to induce potent CD8+ T-cell responses [10-13]. However, strong CD8+ T-cell responses associated with high protection levels is only achieved by using different viral vectors in a prime-boost strategy. Pre-existing immunity, by natural exposure or VLP prime immunization, might reduce the efficacy of a subsequent boost immunization with the same VLP [4]. Moreover, concerns for safety were recently raised from a clinical trial of an Ad5-vectored HIV vaccine, in which excess HIV infections were observed in vera with pre-existing Ad5 antibodies [14]. Both the necessity of prime-boost immunizat! ion with different carriers and the uncertainties about safety profiles could represent hurdles for development of a malaria VLP vaccine; emphasizing the need for alternative delivery platforms.

Previous studies have described a multifunctional carrier system based on *Lactococcus lactis *[15,16], for which prime immunization has been shown not to reduce the efficacy of booster immunizations [17]. By simple hot acid pre-treatment, these bacteria are converted in a non-living particle delivery system with self-adjuvanting properties, called bacterium-like particles (BLPs). BLPs can be simply mixed with antigens to stimulate immune responses. The best stimulation is obtained when the antigen is attached to the particle [17]. Strong, but non-covalent attachment of antigens to the surface of BLPs is mediated by using a lactococcal peptidoglycan binding domain called Protan [18]. Hybrid antigen-Protan fusion proteins can be secreted by a recombinant production system. When the cell-free culture medium is mixed with BLPs, Protan-fusion proteins bind with high affinity. Applications of BLP-based delivery have been successful for influenza [19,20], *Yersinia pestis *[21] and *Streptococcus pneumoniae *[22]. In a previous study, the ability *of Lactococcus lactis *BLPs to elicit systemic antibodies against the *Plasmodium falciparum *merozoite surface antigen 2 was evaluated [23]. In the present study, immune responses and protective efficacy were studied in a murine model following parenteral immunizations with BLPs carrying *Plasmodium berghei *circumsporozoite protein (PbCSP) peptides.

## Methods

### Bacterial strains and growth conditions

Strains and plasmids used in this study are listed in Table 1. *Lactococcus lactis *strains were grown at 30°C in M17 broth (Oxoid) containing 0.5% glucose (w/v) (GM17) and, when necessary, supplemented with chloramphenicol (5 μg/ml) for plasmid selection. P_nisA_-driven gene expression was induced with the culture supernatant of the nisin-producing *L. lactis *strain NZ9700 as described previously [24]. *Escherichia coli *strains were grown in Luria-Bertani (LB) liquid medium or on agar plates at 37°C both supplemented with 100 μg/ml ampicillin. Enzymes and buffers were purchased from New England Biolabs (USA) or Roche (The Netherlands). Electro-transformation of *L. lactis *was carried out as described by Holo and Nes [25] using a Bio-Rad Gene Pulser.

**Table 1:** Bacterial strains and plasmids used in this study

Strain or Plasmid	**Relevant phenotype(s) or genotype(s)**^**a**^	Source/reference
PCR4-PbCSP	Ap^r ^Km^r^, derivative of pCR4Blunt-TOPO (Invitrogen) containing the *Plasmodium berghei *CSP gene (Figure 1)	This study
pPA3	Cm^r^, pNZ8048 derivative containing *c-myc*, the *acmA *Protan (nucleotides 835 to 1492) under control of PnisA, and *usp45*_*ss *_(PA3)	[15]
pPA77	pPA3 derivative producing CSP[Tlong]-Protan	This study
pPA91	Ap^r^, pET32C (Novagen) derivative producing CSP[Tlong]-His	This study
pPA171	pPA3 derivative producing CSP[2xB]-Protan	This study
pPA177	pPA3 derivative producing CSP[Tshort]-Protan	This study
pPA180	Mucosis laboratory collection, used for subcloning	This study
pPA182	Mucosis laboratory collection, used for subcloning	This study
pPA193	Ap^r^, pET32C (Novagen) derivative producing CSP[2xB]-His	This study
pPA197	pPA177 derivative producing CSP[2xB-Tshort]-Protan	This study
pPA198	pPA177 derivative producing CSP[4xB-Tshort]-Protan	This study
*Lactococcus lactis *PA1001	Derivative of NZ9000 lacking *acmA *and *htrA*, allows nisin-inducible expression	[15]
*Escherichia coli *BL21(DE3)	Allows IPTG-inducible expression	Novagen

a) P_nisA_, nisin-inducible promoter; *usp45*_*ss*_, signal sequence of *usp45*

### Plasmid construction and production of antigens

**Figure 1:**
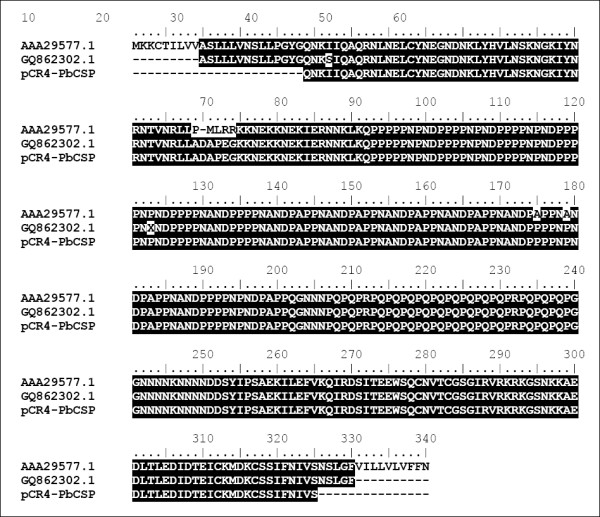
**Alignment of the partial protein sequence encoded by the DNA of the *Plasmodium berghei *strain ANKA CSP gene as cloned in plasmid pCR4-PbCSP (this study) with two sequences of the CSP genes from *P*. *berghei *(Genbank: **AAA29577.1**, [46]) and *P. berghei *strain ANKA (Genbank: **GQ862302.1**, [47])**. Identical residues are shaded black.

PbCSP strain ANKA genomic DNA was isolated as total DNA from infected red blood cells using Genomic Prep™ (Amersham) as described by the manufacturer's instructions. Primers PbCSP.1 (AACGTCTCAC ATGCAAAATA AAATCATCCA AGCCCAAAGG AAC) and PbCSP.2 (CGTCTCAAGC TATTAAAGCT TAAGAATTCC GCTTACAATA TTAAATATAC TTGAAC) were used to amplify the PbCSP gene fragment lacking the parts encoding the signal sequence and GPI anchor. The PCR fragment was cloned into vector pCR4TopoBlunt (Invitrogen, Breda, The Netherlands), resulting in plasmid pCR4-PbCSP. The PbCSP specific parts in this plasmid were sequenced (Figure 1). Plasmid pCR4-PbCSP was used as a template for subsequent cloning in the Protan - and His-tag fusion plasmids. All cloning steps that involved PCR were checked by nucleotide sequence analyses (BaseClear, Leiden, The Netherlands).

**Figure 2:**
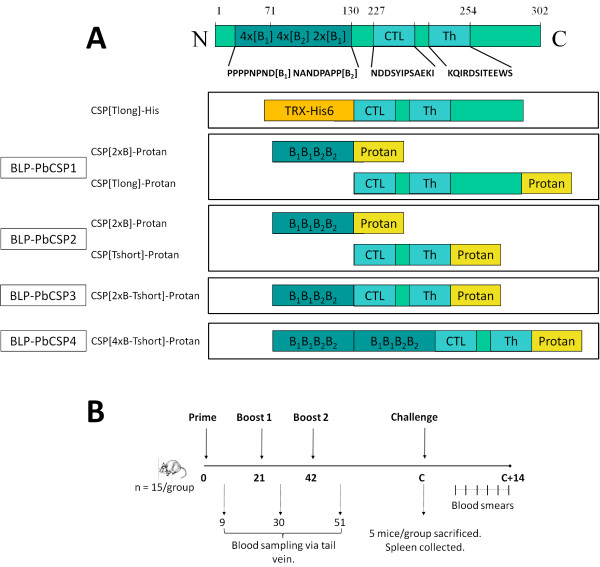
**Schematic representation of BLP-PbCSP constructs and experimental set-up**. (A) Schematic representation of the mature native PbCSP (without GPI anchor) with the B-cell epitopes, the CD8^+ ^and CD4^+ ^T-cell sequences. Formulations used in this study including a CSP-Tlong-His and various BLP-CSP are schematically represented. (B) In several experiments, mice (n = 15/group) were immunized by subcutaneous administration of a prime and two boost injections. Blood samples were collected at day 9, 30 and 51 for serology. Two or three weeks after the second boost, mice were either sacrificed (n = 5/group) or challenged (n = 10/group). Protection was assessed by Giemsa-staining of bloodsmears from day 4 to 14 after challenge.

Plasmid pPA77 (CSP[Tlong]-Protan) encodes the C-terminal part of PbCSP without GPI anchor, but includes CD4^+ ^and CD8^+ ^epitopes, coupled to Protan (Figure 2A). The DNA encoding the CSP-Tlong domain was constructed by ligating a *Nco*I- and *Eco*RI-cleaved PCR-amplified fragment of *P. berghei *CSP1 into the corresponding sites of plasmid pPA3. The CSP-specific fragment was amplified with the primers CSP-ctl.fw1 (CAAACTCCAT GGGAAATGAC GATTCTTATA TCCC) and CSP-ctl.rev1 (CCTGAGCATG CTCGAATTCG GCTTACAATA TTAAATATAC TTGAAC) with plasmid pCR4-PbCSP as template. The resulting plasmid pPA77 was used for electroporation of *L. lactis *PA1001.

Plasmid pPA91 (CSP[Tlong]-His) encodes the same PbCSP fragment as in plasmid pPA77, coupled to a His-tag and a thioredoxin domain (Figure 2A). The DNA encoding the CSP domain was cut from plasmid pPA77 with *Nco*I and *Eco*RI and ligated in the corresponding sites of plasmid pET32C. For intracellular production of protein via IPTG induction, plasmid pPA91 was transferred to *E. coli *strain BL21(DE3).

Plasmid pPA171 (CSP[2xB]-Protan) specifies a fusion between selected B-cell epitopes (32 amino acids) of the *P. berghei *CSP protein ([PPPPNPND]_2x_-[NANDPAPP]_2x_) and Protan (Figure 2A). The DNA encoding the B-cell domain was produced by annealing four primers based on *L. lactis *codon usage. A PCR without template was performed with primers 273 (CGGTCTCACA TGGATATCGG AATTCCTCCA CCTCCAAATC CTAATGATCC ACCTC), 274 (GATCATTAGC ATTATCATTA GGATTAGGTG GAGGTGGATC ATTAGGATTT), 275 (TAATGATAAT GCTAATGATC CAGCTCCACC TAACGCAAAT GACCCTGCTC) and 276 (CGGTCTCTAA TTCCTGGAGG AGCAGGGTCA TTTGCGTTA). A second PCR with only the flanking primers 273 and 276 was performed on the product of the first PCR. The resulting 134 bp product was ligated in the pCR^®^4Blunt-TOPO vector and sequenced. An *Eco*31I cleaved insert from this plasmid, possessing *Nco*I and *Eco*RI overlapping ends, was then transferred into the corresponding sites of vector pPA3. The resulting plasmid pPA171 was used for electroporation of *L. lactis *PA1001.

Plasmid pPA177 (CSP[Tshort]-Protan) specifies a fusion between the CD8^+ ^and CD4^+ ^T-cell epitopes of the *P. berghei *CSP protein and Protan. Compared to the CSP[Tlong]-Protan construct, the CSP[Tshort]-Protan construct lacks the amino acids C-terminal of the predicted CD4^+ ^T-cell epitope (Figure 2A). The DNA encoding the CSP[Tshort] domain was constructed by a PCR without template with four partly overlapping primers 240 (CGGTCTCACA TGGATATCGG AATTCAAAAT GATGATTC), 241 (GGAATTCAAA ATGATGATTC ATATATTCCA TCTGCTGAAA AAATTTTAGA ATTTG), 242 (CCATTCTTCT GTAATACTAT CACGAATTTG TTTAACAAAT TCTAAAATTT TTTCAG) and 243 (CGGTCTCTAA TTTGTGACCA TTCTTCTGTA ATACTATCAC), representing the desired CSP[Tshort] sequence with *L. lactis *optimized codon usage. The product was cloned in the pCR^®^4Blunt-TOPO vector. An *Eco*31I-cleaved fragment of the resulting vector containing the insert, resulting in *Nco*I and *Eco*RI overhanging ends, was ligated into the corresponding sites of plasmid pPA3, thereby replacing the *myc*-epitope present in pPA3. The resulting plasmid pPA171 was used for electroporation of *L. lactis *PA1001.

Plasmid pPA193 (CSP[2xB]-His) specifies a fusion between selected B-cell epitopes as in plasmid pPA171, coupled to a His-tag and a thioredoxin domain. The *Eco*31I-cleaved fragment that was used in the construction of pPA177 was ligated in plasmid pPA180, resulting in plasmid pPA182, now containing DNA encoding the B-cell epitope with a C-terminal His-tag. A *NcoI *and *Hind *III cleaved fragment of pPA182 was ligated in the corresponding sites of plasmid pET32C, resulting in plasmid pPA193. For intracellular production of protein via IPTG induction, plasmid pPA193 was transferred to *E. coli *strain BL21(DE3).

Plasmid pPA197 (CSP[2xB-Tshort]-Protan) specifies a fusion between selected B-cell epitopes as in plasmid pPA171, the CSP Tshort epitope (CTL and Th) and Protan, and was constructed by ligating an *Eco*RI-cleaved fragment of pPA171, containing the B-cell epitope, into the *EcoRI *site of pPA177 in front of the Tshort - Protan fusion. The ligation mix was used for electroporation of *L. lactis *PA1001.

Plasmid pPA198 (CSP[4xB-Tshort]-Protan) specifies a fusion between two selected B-cell epitopes as in plasmid pPA171, the CSP Tshort epitope (CTL and Th) and Protan, and was constructed by ligating two sequential *Eco*RI-cleaved fragments of pPA171, containing the 2xB cell epitope, into the *EcoRI *sites of pPA177. The resulting plasmid pPA198 was used for electroporation of *L. lactis *PA1001.

### Preparation of BLP-PbCSP formulations

CSP[2xB]-His and CSP[Tlong]-His were produced by *E. coli *using IPTG induction and purified by His-tag isolation for coating of ELISA plates. Purified CSP[Tlong]-His was also used for immunizations after addition of incomplete Freund's adjuvant (IF A).

The plasmids pPA171, pPA77 and pPA177 were used to express and secrete the recombinant fusion proteins CSP[2XB]-Protan, CSP[Tlong]-Protan and CSP[Tshort]-Protan, respectively (see Figure 2A). BLP-PbCSP were prepared as described elsewhere [16]. Briefly, BLPs were obtained by boiling freshly grown *L. lactis *NZ9000 in 0.6 M trichloroacetic acid (pH = 1) for 30 min, followed by extensive washing with phosphate buffered saline (PBS). Production of the antigen-Protan fusions was induced by addition of nisin to cultures of the appropriate plasmid carrying *L. lactis *PA1001 strains. Culture supernatants containing the Protan-fusion proteins were 10 times concentrated by ultrafiltration using a VivaFlow system (VivaFlow200, 10,000 Da cut-off, Vivascience, Hannover, Germany). Binding of antigens was achieved by mixing the concentrates with BLPs under gentle agitation for 30 min at room temperature, followed by extensive washing with PBS.

BLP-PbCSP1 was produced as follows: concentrated supernatant of a CSP[Tlong]-Protan producing *L. lactis *culture was bound to BLPs on a blood suspension mixer for 30 min at room temperature. After washing with PBS, CSP[2xB]-Protan was bound to the BLP-CSP[Tlong]-Protan using the same method. Next, the pellet was washed and re-suspended in PBS, resulting in a BLP-PbCSP containing both antigens on a single particle. BLP-PbCSP1 contained approximately 5 μg of CSP[Tlong]-Protan and 20 μg CSP[2xB]-Protan per dose. The same approach was used for BLP-PbCSP2, which was produced by first binding the CSP[Tshort]-Protan to the BLP followed by washing and binding of the CSP[2xB]-Protan antigen. BLP-PbCSP2 contained 20 μg of CSP [Tshort]-Protan and 20 μg CSP[2xB]-Protan per dose. BLP-PbCSP3 and BLP-PbCSP4 contained approximately 45 μg of CSP[2xB-Tshort]-Protan per dose. The amount of bound antigen-Protan was estimated using Coomassie brilliant blue stained gels and compared to BSA protein standards. BLP-PbCSP inocula each contained 2.5 × 10^9 ^BLPs in 100 μl PBS. To formulate the control for BLP-PbCSP1, 10 μg CSP[Tlong]-His was emulsified in IFA (Difco Laboratories, Michigan, U.S.A) in final volume of 100 μl.

### Immunization, sample collection and challenge

All animal experiments were performed with approval of the Animal Experimentation Committee of the University of Nijmegen, The Netherlands (DEC2002-89 and DEC2005-70). Female BALB/c mice (six week old) were purchased from Charles River (Germany) and received water and food ad libitum. Mice (n = 15) were immunized subcutaneously by injection of 100 μl of CSP formulation or PBS, divided into both flanks. Complete immunization consisted of a prime and two boosts injections given at three-week intervals. Two weeks after the last administration, blood samples were collected from the retro-orbital plexus and supplemented with heparin. After centrifugation, plasma was stored at -20°C until use. Immunized mice were either challenged by bites of infected mosquitoes (n = 10) or sacrificed for IFN-γ ELISPOT assay (n = 5) as described below. Challenge of immunized and naive mice was performed by bites of five to eight infected *P. berghei *mosquitoes two to three weeks after the second boost injection. Parasitaemia was determined every other day from day 4 to day 14 by Giemsa-staining of blood smears. Mice that developed parasitaemia were sacrificed on day 12 for ethical reasons. Mice with negative blood smears on day 14 after challenge were considered fully protected. An overview of the experiment is presented in Figure 2B.

### Measurement of IFN-γ production

Two weeks after the last immunization, spleens of non-challenged mice were collected in culture medium (DMEM high glucose, containing Glutamax and supplemented with 10% FCS and Penicillin/Streptavidin). Spleen cell suspensions were prepared by mechanical dispersion and processed through a 70 μm Nylon cell strainer (BD Falcon). Red blood cells were lysed by incubation with ACK buffer (NH_4_Cl 0.83%, KHCO_3 _1 mM, EDTA 0.1 mM, pH = 7.4) for 5 min. IFN-γ production was measured using the mouse IFN-γ ELISPOT kit from BD Biosciences (Erembodegem, Belgium), according to the manufacturer's instruction. Splenocytes were plated at a density of 4 × 10^5 ^or 10^6 ^cells per well and stimulated with the CSP peptide sequence of the CD4+ eptitope KQIRDSITEEWS or the CD8^+ ^epitope SYIPSAEKI (synthesized by Sigma Genosys). Stimulation with 4 μg/ml PHA (Sigma-Aldrich) served as positive control for the assay. The CSP-Tlong-His formulation (Figure 2A), containing CD4+ and CD8+ T-cell epitopes previously shown to induce IFN-γ responses [26-28], served as a positive control. Plates were incubated for 16 hr at 37°C - 5% CO_2 _prior to IFN-γ staining. Spots were counted using the A. EL. VIS (Automated ELISA Spot Assay Video Analysis Systems), Eli. Scan and Eli. Analyse software (Sanquin, Amsterdam, the Netherlands). In all experiments, polyclonal stimulation with PHA showed in all immunized and naïve mice equally high IFN-γ response compared to unstimulated conditions (p < 0.05).

### Measurement of anti-CSP antibodies

Specific anti-CSP IgG concentrations were determined by enzyme-linked immuno sorbent assay (ELISA). Briefly, high-binding capacity microtitre plates (Greiner, Alphen aan de Rijn, the Netherlands) were coated with CSP[2xB]-His - (0.2 μg/well) in 0.05 M carbonate buffer (pH = 9.6) overnight at 4°C. The plates were washed with PBS - 0.02% Tween 20 (pH = 7.4), then incubated for 1 hr with 1% BSA in PBS/Tween. Diluted sera were added to the plates in three-fold dilutions and incubated for 2 hr at room temperature. After washing, the alkaline phosphatase secondary antibody directed to mouse IgG-Fc (Sigma-Aldrich, Zwijndrecht, The Netherlands) was incubated for 1.5 hr at a dilution 1:5,000. Colorimetric reaction was obtained by addition of p-nitrophenyl phosphate substrate (Sigma-Aldrich) diluted in 0.05 M carbonate buffer (pH = 9.6) supplemented with 1 μM MgCl_2_. The enzymatic reaction was stopped with NaOH and read at 405 nm. The CSP specific IgG concentrations were calculated by comparisons with a calibration curve obtained with purified mouse IgG (Sigma-Aldrich).

### Statistical analysis

Comparisons between groups were performed by one-way ANOVA (Kruskas-Wallis test) or by a Mann-Whitney *U *test using PRISM software version 5.0 (Graphpad, San Diego, CA). p < 0.05 was considered statistically significant.

## Results

### BLP-PbCSP1 induces parasite-specific antibodies but no IFN-γ response

**Figure 3:**
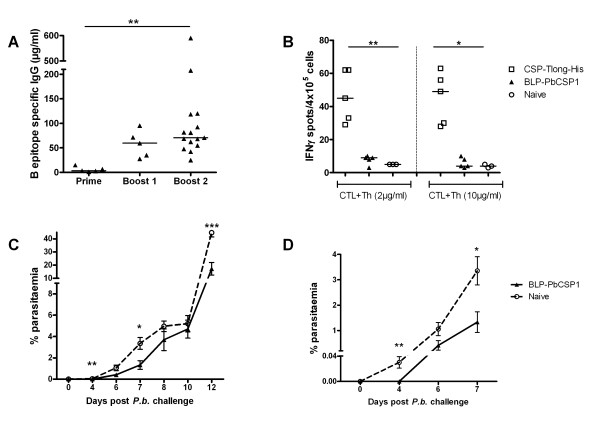
**Immune responses and protection BLP-PbCSP1**. BLP-PbCSP1 induced (A) IgG antibodies against B-cell epitopes ([PPPPNPND]_2x_-[NANDPAPP]_2x_) and (B) IFN-γ response against CTL (SYIPSAEKI) and Th (KQIRDSITEEWS) *P. berghei *CSP epitopes. (C + D) Post-challenge parasitaemia percentages and pre-patency upon detailed view of the first seven days of infection. Median are presented on all plots with individual values. Error bars represent SEM. * = p < 0.05, ** = p < 0.01, *** = p < 0.001.

BLP-PbCSP1 was produced by expression of both CSP B- and T-cell epitopes in two different Protan fusion products (Figure 2A). Following subcutaneous immunization, B-cell-epitope specific IgG levels were determined by ELISA nine days after prime (day 9) and each boost injection (day 30 and 51). The prime injection induced low levels of B-cell-epitope specific IgG levels that significantly increased after the first boost injection (p = 0.008) and remained at similar levels upon the second boost (Figure 3A). Prior to challenge, IFN-γ response was assessed by ELISPOT in five of the 15 BLP-PbCSP1 immunized mice. However, no IFN-γ response could be observed after stimulation with CSP epitopes (Figure 3B).

### Partial protection by BLP-PbCSP1

Next, protection in 10 BLP-PbCSP1 immunized mice was assessed by performing a mosquito challenge three weeks after the second boost. At day 14 post-challenge, BLP-PbCSP1 showed complete protection in 40% (4/10) of the mice (Table 2) and the development of blood-stage parasites in immunized unprotected mice did not differ from naive mice (Figure 3C). However, the pre-patent period of unprotected mice was significantly extended for two days compared to naive mice suggestive for partial immunity (Figure 3D).

**Table 2:** Protection by BLP-PbCSP1, 2 and 4 upon *Plasmodium berghei *sporozoite challenge

		No. protected/No. challenged	% protection
Exp 1	BLP-PbCSP1	4/10	40%
	Naive	0/10	0%

	BLP-PbCSP1	3/10	30%
Exp 2	BLP-PbCSP2	7/10	70%
	Naive	0/10	0%

	BLP-PbCSP2	6/10	60%
Exp 3	BLP-PbCSP4	9/9	100%
	Naive	1/10	10%

### Improved immune responses and protection by optimized BLP-PbCSP2

**Figure 4:**
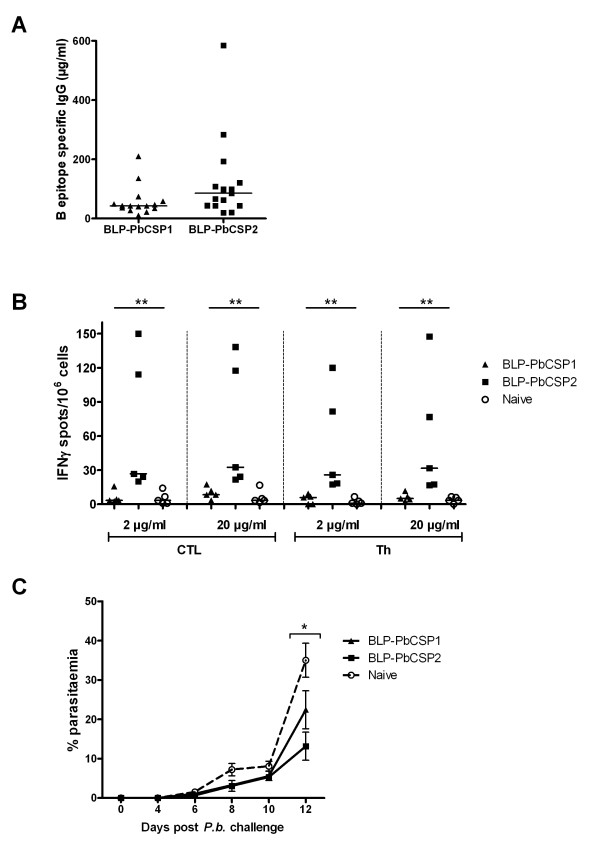
**Immune responses and protection BLP-PbCSP2**. (A) BLP-PbCSP2 IgG levels against B-cell epitopes ([PPPPNPND]_2x_-[NANDPAPP]_2x_) compared to BLP-PbCSP1. (B) BLP-PbCSP2 induced IFN-γ response against CTL (SYIPSAEKI) and Th (KQIRDSITEEWS) *P. berghei *CSP epitopes. (C) Post-challenge parasitaemia percentages. Medians are presented on all plots with individual values. Error bars represent SEM. * = p < 0.05, ** = p < 0.01.

To further improve BLP-PbCSP, codons were optimized for higher production in *L. lactis *and the length of the non-immunogenic sequence of the T-cell epitope was shortened. Optimization resulted in a new fusion protein BLP-PbCSP2 with CSP[Tshort]-Protan bound to BLP in equal amounts as the CSP[2xB]-Protan (Figure 2A). Following a prime and two boost injections (Figure 2B), BLP-PbCSP2 immunized mice developed slightly higher concentrations (p = 0.05) of B-cell epitope specific IgG levels compared to BLP-PbCSP1 (Figure 4A). Unlike BLP-PbCSP1 however, ELISPOT IFN-γ levels were high in response to both CTL and Th peptides (Figure 4B). Thus optimization of the BLP-PbCSP1 resulted in BLP-PbCSP2 with slightly higher humoral and enhanced cellular responses. Challenge of BLP-PbCSP1 and BLP-PbCSP2 immunized mice was performed two weeks after the second boost. Complete protection was observed by day 14 post-challenge in 30% (3/10) of BLP-PbCSP1 and in 70% (7/10) of BLP-PbCSP2 immunized mice (Table 2). Parasitaemia of the BLP-PbCSP2 immunized mice was significantly lower on day 12 in unprotected immunized mice (p = 0.02) compared to naive mice (Figure 4C).

### Stronger immune response and 100% protection by BLP-PbCSP4

**Figure 5:**
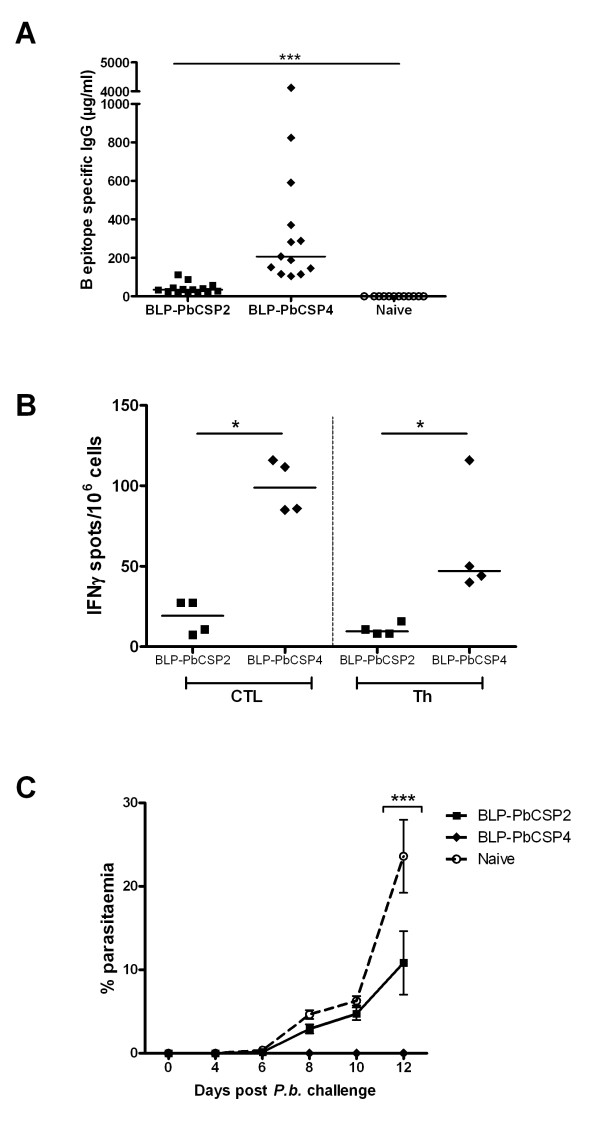
**Immune responses and protection BLP-PbCSP4**. BLP-PbCSP4 induced (A) IgG levels against B-cell epitopes ([PPPPNPND]_2x_-[NANDPAPP]_2x_) and (B) IFN-γ response against CTL (SYIPSAEKI) and Th (KQIRDSITEEWS) *P. berghei *CSP epitopes compared to BLP-PbCSP2. (C) Post-challenge parasitaemia of unprotected BLP-PbCSP4 and two immunized mice. Medians are presented on all plots with individual values. Error bars represent SEM. * = p < 0.05, *** = p < 0.001.

Finally, to further improveme humoral responses induced by BLP-PbCSP, B-cell and T-cell epitopes were fused to formulate BLP-PbCSP3 and B-cell epitopes were duplicated in BLP-PbCSP4 (Figure 2A). Anti-B-cell epitope IgG responses generated by BLP-PbCSP4 were marginally higher compared to BLP-PbCSP3 (145 μg/ml and 104 μg/ml respectively, data not shown). Immunization with BLP-PbCSP4 induced significantly higher IgG antibody levels against B-cell epitopes as compared to BLP-PbCSP2 immunized and naïve mice (p < 0.0001), (Figure 5A). Furthermore, significantly higher CTL- and Th-specific IFN-γ responses were observed by splenocytes from BLP-PbCSP4 (p = 0.03) compared to BLP-PbCSP2 immunized mice (Figure 5B). Following challenge (two weeks after the second boost), complete protection was observed in 60% (6/10) of BLP-PbCSP2 and in 100% (9/9) of BLP-PbCSP4 immunized mice (Table 2). In line with the previous experiment, unprotected mice immunized with BLP-PbCSP2 showed a significant lower parasitaemia (p = 0.03) at day 12. All mice immunized with BLP-PbCSP4 developed sterile immunity (Figure 5C). Thus, optimization of the BLP-PbCSP formulations resulted in high T- and B-cell responses that provided complete protection in all *P. berghei *immunized mice.

## Discussion

The present study reports 100% protection against *P. berghei *malaria induced by subunits of PbCSP using bacterium-like particles (BLPs) as self-adjuvanting delivery system. Following subcutaneous immunization, BLP-PbCSP formulations are able to trigger both specific IgG antibodies and IFN-γ response to CD4+ and CD8+ T-cell epitopes. Both humoral and cellular responses have been shown to be relevant for protection against malaria parasites in different murine models [29-32]. Identification and use of specific T- and B-cell epitopes has been valuable for understanding the protective immune response induced by CSP-based constructs [28,32-34]. Immunization with virally vectored *P. berghei *CSP shows low immune responses and protection levels following prime-boost regimen with a single carrier [10-13]. However, immune responses and protection rates are high when different viral vectors for *P. berghei *or *Plasmodium yoelii *CSP are used [10-13,35]. Seemingly, the benefits of VLP-based carrier combinations for induction of high protective efficacy apply to several *Plasmodium *species. Similarly, prime-boost *P. berghei *CSP immunizations with *Salmonella *or *Bordetella-based *carriers show lower protective efficacy compared to the combination of both carriers [36]. The current data show that homologous prime-boosting with *L. lactis *BLP-PbCSP is sufficient to induce sterile protection, illustrating the potency of this platform for effec! tive del ivery of malaria epitopes.

The several epitope modifications explored to improve immune responses of BLP-PbCSP constructs included codon optimization, reduction of the non-immunogenic part in the T-cell epitopes as well as fusion of B- and T-cell epitopes. As previously shown, codon optimization can improve adequate production of malaria antigens [37]. The improved cellular responses reported here cannot be clearly explained by either codon optimization or modification of T-cell epitopes. As for humoral responses, CD4+ helper T cells contribute to B-cell responses and even more so are prerequisite to IgG production. Tam et al. reported highest antibodies titres and highest protection levels by tandemly connected T- and B-cell epitopes of PbCSP [38]. Accordingly, improved T helper responses may indirectly account for the somewhat increased IgG responses to BLP-PbCSP2. As presented in Figure 3, immunization with BLP-PbCSP1 induces high levels of B-cell epitope specific IgG but no CTL/Th specific IFN-γ response, resulting in low protection level and significant delayed parasitemia. While immunization with BLP-PbCSP2 merely induces slightly higher levels of B-cell epitope specific IgG, the significantly higher CTL/Th specific IFN-γ response associates with higher protection level and further delayed parasitaemia. Eventually, BLP-PbCSP4 induces the highest levels of humoral and cellular responses but also complete protection. Clearly, induction of specific antibodies is sufficient to induce a minimal protective efficacy. For complete protection however, both potent humoral and cellular responses are apparently required. Whether contribution, if any, of the Th response to humoral response leads to qualitatively superior specific IgG antibodies is an interesting consideration to explore in future studies.

Moreover, CSP contains several epitopes that have been shown to induce B-cell [32,39,40] as well as CD4+ and CD8+ T-cell [30,41,42] protective immune responses. The present study clearly further illustrates that modifications in both T- and B-cell CSP epitopes can have major effects on immune responses and protective efficacy as shown before [38]. In addition, there is increasing evidence of the protective role of non-CSP pre-erythrocytic malaria antigens [43-45]. Immunization studies with some recently identified candidates could be performed using the *L. lactis *BLP-carrier whose immuno-modulating properties that induce local and systemic immune responses [17,19,21], are an asset for efficient protection studies with potentially multiple antigen peptides. Recent launch of a phase I clinical study with FluGEM™, an influenza vaccine with *L. lactis *BLP as delivery platform, paved the way towards vaccines delivery by this non-living and non-genetically modified carrier. Eventually, BLPs derived from the food-grade bacterium *L. lactis *could also be evaluated as a delivery system for a malaria vaccine.

In conclusion, immunizations with BLP-PbCSP formulation containing B- and T-cell epitopes induce strong humoral and cellular responses that result in complete protection in mice. *L. lactis *BLPs are potentially a delivery system for the development of a safe and affordable malaria vaccine with high protective efficacy.

## Abbreviations

BLP: Bacterium-like particle; (Pb)CSP: (*Plasmodium berghei*) circumsporozoite protein.

## Competing interests

The authors declare that they have no competing interests.

## Authors' contributions

KN performed data analysis and drafted the manuscript. MvR, KL, CH and RS helped to draft the manuscript. GvG carried out mosquito challenge. MvR and SA carried out the study conceived and coordinated by KL, CH and RS. All authors read and approved the final manuscript.
